# Grain number responses to pre-anthesis dry matter and nitrogen in improving wheat yield in the Huang-Huai Plain

**DOI:** 10.1038/s41598-018-25608-0

**Published:** 2018-05-08

**Authors:** Jianzhao Duan, Yapeng Wu, Yi Zhou, Xingxu Ren, Yunhui Shao, Wei Feng, Yunji Zhu, Yonghua Wang, Tiancai Guo

**Affiliations:** 1 National Engineering Research Centre for Wheat, State Key Laboratory of Wheat and Maize Crop Science, Henan Agricultural University, #62 Nongye Road, Zhengzhou, Henan 450002 PR China; 2 Wheat Research Center of Henan Academy of Agricultural Sciences, Zhengzhou, 450002 China

## Abstract

Wheat yield components vary between different ecological regions and yield levels. Grain number responses to pre-anthesis dry matter (DM) and nitrogen (N) in increasing yield were always investigated in spike organs, neglecting the effect of non-spike organ nutrition or overall distribution. This paper determined the relationships between grain number and pre-anthesis DM and N in spike and non-spike organs under different yield levels, with using two sorts of field experiments (different water-nitrogen modes and cultivation management patterns) from 2012–2015 in Huang-Huai plain. The results indicated that improving yield under yield of <7500 kg ha^−1^ depends on increasing grain number per spike (GNs) or spike number (SN) or both, increased yield under higher yield of >7500 kg ha^−1^ mainly depends on GNs. GNs showed significant positive relationships with above-ground DM accumulation from jointing to anthesis under high or low yield levels. Rapid DM growth in spring achieves higher GNs. Spike and non-spike DM and N contents both demonstrated strong positive relationships with GNs, spike DM distribution also shows a positive correlation, but spike N distribution ratio show negatively correlation with GNs. Improved N distribution in non-spike organs and DM partition in spike organs conduce to increasing GNs.

## Introduction

In recent years, the burgeoning world population means that a substantial increase in grain yield is needed^1,2^. In wheat (Triticum aestivum L.) production, yield is an extremely complex trait, determined by agricultural manipulation and breeding attributes^1,3^. Research efforts focusing on physiological characteristics of wheat and improved production technologies aimed at breeding of new high-yielding wheat varieties are therefore necessary^4,5^. Wheat yield is determined by its components such as spike number per unit area (SN), grain number per spike (GNs) and thousand grain weight (TGW)^4,6,7^. Increased coordination between yield components is required to improve yield potential. Some studies in America and Europe have shown that both SN and GNs determine grain number per unit area (GNa)^8–10^, but the above three indicators were analyzed respectively in China. GNa and TGW are determined at different periods of growth. While GNa is largely dependent on floret set at pre-anthesis, TGW relies on post-anthesis grain-filling^11^. Yield potential can be improved by increasing GNa or TGW^2,8,9,12^, however, during the past century, increased yield have been largely attributed to increases in GNa^13,14^, frequently relating sink limitations in wheat^15–18^. With improvements in breeding technology, large spike varieties have been widely bred, and therefore, such the characters of sink may have some variation in increased grain yield. In addition, improvements in planting technology and increased sowing quantity have caused wheat population spikes to reach saturation, suggesting that improved yield is perhaps dependent on increased GNs. Nevertheless, the optimal approach to increase yield in modern cultivars remains unclear, as does the current role of GNs in increasing wheat yield.

The month before flowering is a critical period of floret differentiation in wheat, determining the GNs^19^. The process of floret primordia differentiation into fertile florets is dependent on nutrient supplements, with a strong positive relationship between floret survival and aboveground DM (especially spike DM) at anthesis^20,21^. Grain formation is therefore dependent on the pre-anthesis growth status^14,22^. Previous studies also revealed a closely relationship between spike DM at anthesis and grains number in wheat^23,24^. This suggested that adequate nutrition is the foundation for achieving higher grain number.

Nitrogen (N) is an important constituent of chlorophyll and metabolic enzyme activities, and one of the main limiting factors of crop production^25,26^. Previous studies have revealed a reduction in GNa with N deficiency before anthesis^27^, and a strong correlation between GNa and spike N content at anthesis^20^. Furthermore, Abbate *et al*.^28^ revealed that GNa is more strongly related to spike N than spike DM accumulation at anthesis. However, others confirmed the strongly relationship between GNa and spike DM, and further considered that spike DM dominated the determination of spike N to GNa^20,29^. To sum up, above-mentioned studies showed the importance of N to GNa. However, the above results mainly focus on the relationship between spike nutrient matter and grain formation, with few studies documenting the partition of nutrients in different organs. For example, Demotes-Mainard *et al*.^30^ studied changes in the proportion of DM and N in the spikes, but not the relationship between GNa and nutrient matter partitioning during spike growth. In addition, although previous research suggests that GNa is related to both spike N and DM, few studies have examined the role of non-spike organ N or DM during the process of floret differentiation and grain formation.

Huang-Huai Plain is the main producing area of winter wheat in China. With improvements in wheat varieties and soil fertility, effective spikes become saturated (over 6, 750, 000 spike ha^−1^), and the TGW reaches levels of 40~52 g. In the current experimental region, the long young ear differentiation period (160~170d) and more differentiated florets (about 180 spikes^−1^) suggest that GNs has great potential for improvement. In order to illuminate the contribution of GNs to yield and the effect of dry matter and nitrogen on GNs, we carried out a series of experiments in the Huang-Huai plain, China. The aims were to: (i) identify the main factors of yield components in increasing grain yield under different production levels (>7500 and <7500 kg ha^−1^); (ii) describe the relationship between aboveground DM and GNs during pre-anthesis at each production level; (iii) clarify the characteristics of spike and non-spike organ DM in relation to GNs during spike growth; and (IV) characterize the relationship between spike and non-spike organ N in terms of GNs during spike growth before anthesis.

## Results

### Changes in yield components and the impact on grain yield

**Figure 1:**
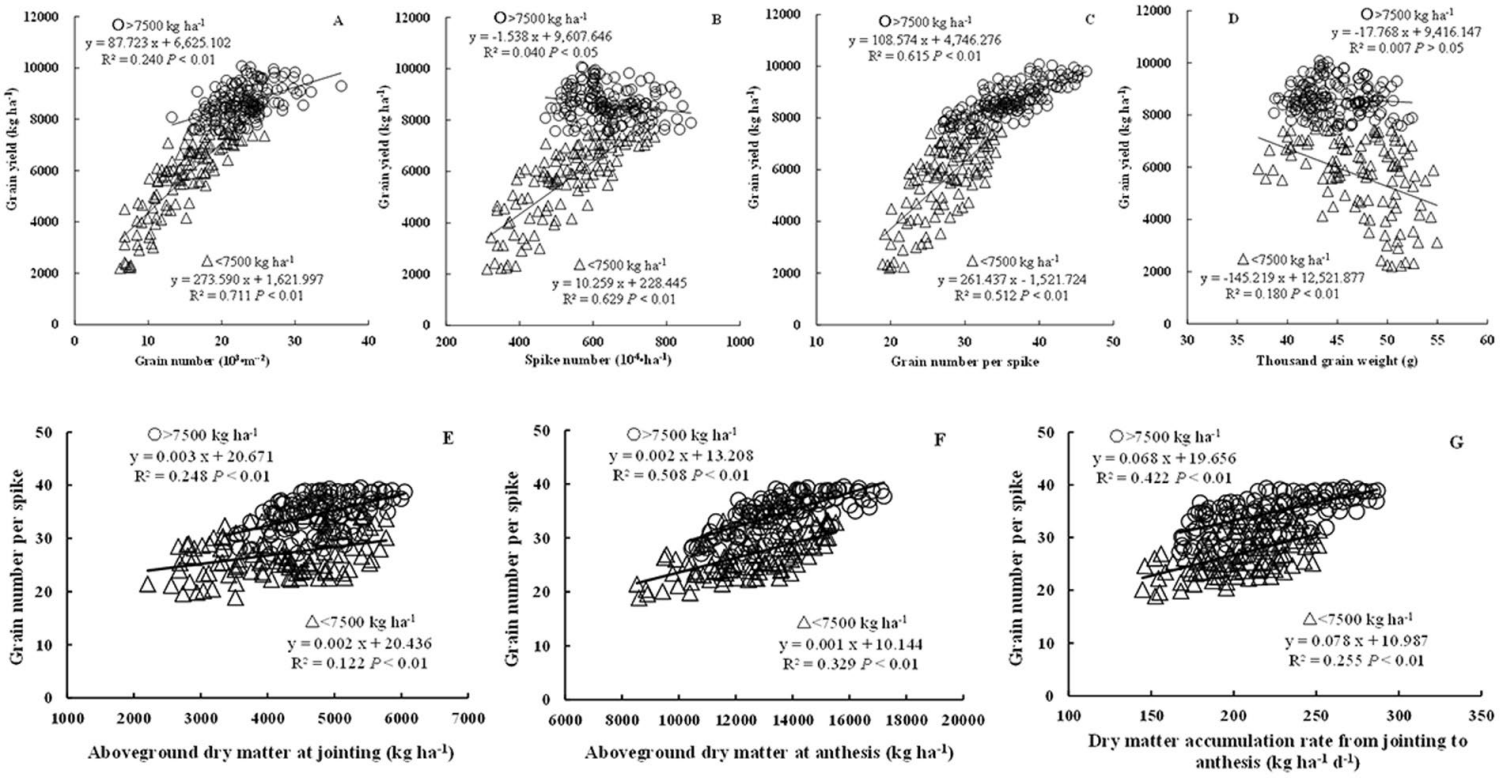
Relationship between grain yield and yield components and relationship between grain number per spike and pre-anthesis dry matter and accumulation rates of aboveground dry matter at different phases before anthesis.

Yield components included SN, GNs, and TGW. These components were not independent, but rather, correlated in a complex manner, combining to generate yield. Figure 1(A–D) showed varying relationships between yield components and yield. Above or below 7500 kg ha^−1^, relationship between yield components and yield showed larger differences, spike number showed significant negative correlation with grain yield when yield was above 7500 kg ha^−1^, but significant positive correlation under yield levels below 7500 kg ha^−1^; thousand grain weight showed poor correlation with grain yield under yield levels above 7500 kg ha^−1^, but significant negative correlation under yield below 7500 kg ha^−1^. And 7500 kg ha^−1^ could be as the boundary of analyzing relationships between each yield components and yield. Here we define yield of <7500 kg ha^−1^ as low yield levels and yield of >7500 kg ha^−1^ as higher yield levels. Both the two groups yield showed significantly positive relationship between GNa and yield (R^2^ = 0.240, >7500 kg ha^−1^; R^2^ = 0.711, <7500 kg ha^−1^), while the R^2^ of higher yield levels was lower than that of low yield levels. GNa could be further divided into two sub-components, i.e., SN and GNs. Low yield levels generated a significantly positive linear relationship between yield and SN (R^2^ = 0.629), while the higher yield level was poor negative correlation. Both the two yield levels resulted in significantly relationship between yield and GNs (R^2^ = 0.615, P < 0.01 for >7500 kg ha^−1^; R^2^ = 0.512, P < 0.01 for <7500 kg ha^−1^). The above higher R^2^ of SN than GNs indicated that SN was primary in increasing yield under low yield levels compared to GNs. Under higher yield levels, coefficient of determination between GNs and yield was higher than that between GNa and yield (Fig. 1A–C). So we mainly studied the effect of GNs on increasing yield. The correlation between TGW and yield was poor at higher yield levels, and significantly negative at low yield levels (*R*^2^ = 0.180, p < 0.01). These results suggest that improvements in yield of <7500 kg ha^−1^ are attributable to increases in both GNs and SN, the latter being the primary factor. However, in contrast, increases in yield of >7500 kg ha^−1^ are mainly dependent on a marked increase in GNs.

### Relationships between GNs and pre-anthesis DM and DM growth rate

The difference between the whole data set and subsets representing different yield levels was noticeable in terms of the relationship between GNs and pre-anthesis aboveground DM at different stages. As shown in Table 1, GNs was significantly positively related to aboveground DM at different growth stages (*r* = 0.529~0.654, *P* < 0.01) when the experimental data was pooled. However, the relationships between DM and GNs differed when subsets of the two yield rates were applied. Coefficients of determination were significant except at wintering and turning green at higher yield levels, and at all stages at low yield levels. In addition, higher coefficients of determination (R^2^ = 0.248~0.508) were observed at higher yield levels than the low (R^2^ = 0.122~0.329) from jointing to anthesis (Table 2 and Fig. 1E and F). Coefficients of determination increased with growth from jointing to anthesis regardless of whether the data set was pooled or divided into subsets, reaching a maximum at anthesis (R^2^ = 0.508 at higher yield levels and R^2^ = 0.329 at the low, Fig. 1F). From the above, higher aboveground DM before anthesis was important in increased GNs, especially at higher yield levels.

**Table 1:** Pearson’s correlation between grain number per spike and aboveground dry matter at different stages.

Production levels (kg ha^−1^)	Wintering stage	Turning green	Jointing	Booting	Heading	Anthesis
>7500	0.067	0.111	0.498^**^	0.595^**^	0.699^**^	0.713^**^
<7500	0.546^**^	0.493^**^	0.349^**^	0.524^**^	0.543^**^	0.574^**^
all	0.556^**^	0.542^**^	0.529^**^	0.549^**^	0.621^**^	0.654^**^

Notes: n = 228; *Significant at the 0.05 probability level; **Significant at the 0.01 probability level.

**Table 2:** Pearson’s correlation between grain number per spike and dry matter accumulation rates at different stages.

Production levels (kg ha^−1^)	Wintering to Turning green	Turning green to Jointing	Jointing to Booting	Booting to Heading	Heading to Anthesis	Jointing to Anthesis
>7500	0.110	0.316^**^	0.445^**^	0.576^**^	0.510^**^	0.650^**^
<7500	0.125	0.259^**^	0.431^**^	0.501^**^	0.402^**^	0.505^**^
all	0.067	0.280^**^	0.347^**^	0.388^**^	0.369^**^	0.584^**^

Notes: n = 228; *Significant at the 0.05 probability level; **Significant at the 0.01 probability level.

Growth rates at different developmental stages affected aboveground DM accumulation. GNs was significantly positively related to DM growth rates at all stages expect wintering to turning green, regardless of whether the data was pooled or divided into subsets (Table 2). Coefficients of determination were higher (r = 0.316~0.650) at higher yield levels than the low (r = 0.259~505) from turning green to anthesis, reaching a maximum (r = 0.50~0.65) from jointing to anthesis (i.e. stem elongation stage), especially at higher yield levels. As shown in Fig. 1G, increasing DM accumulation from jointing to anthesis was important in increasing GNs.

### Relationships between GNs and the dry weights of spike and non-spike organs before anthesis

**Figure 2:**
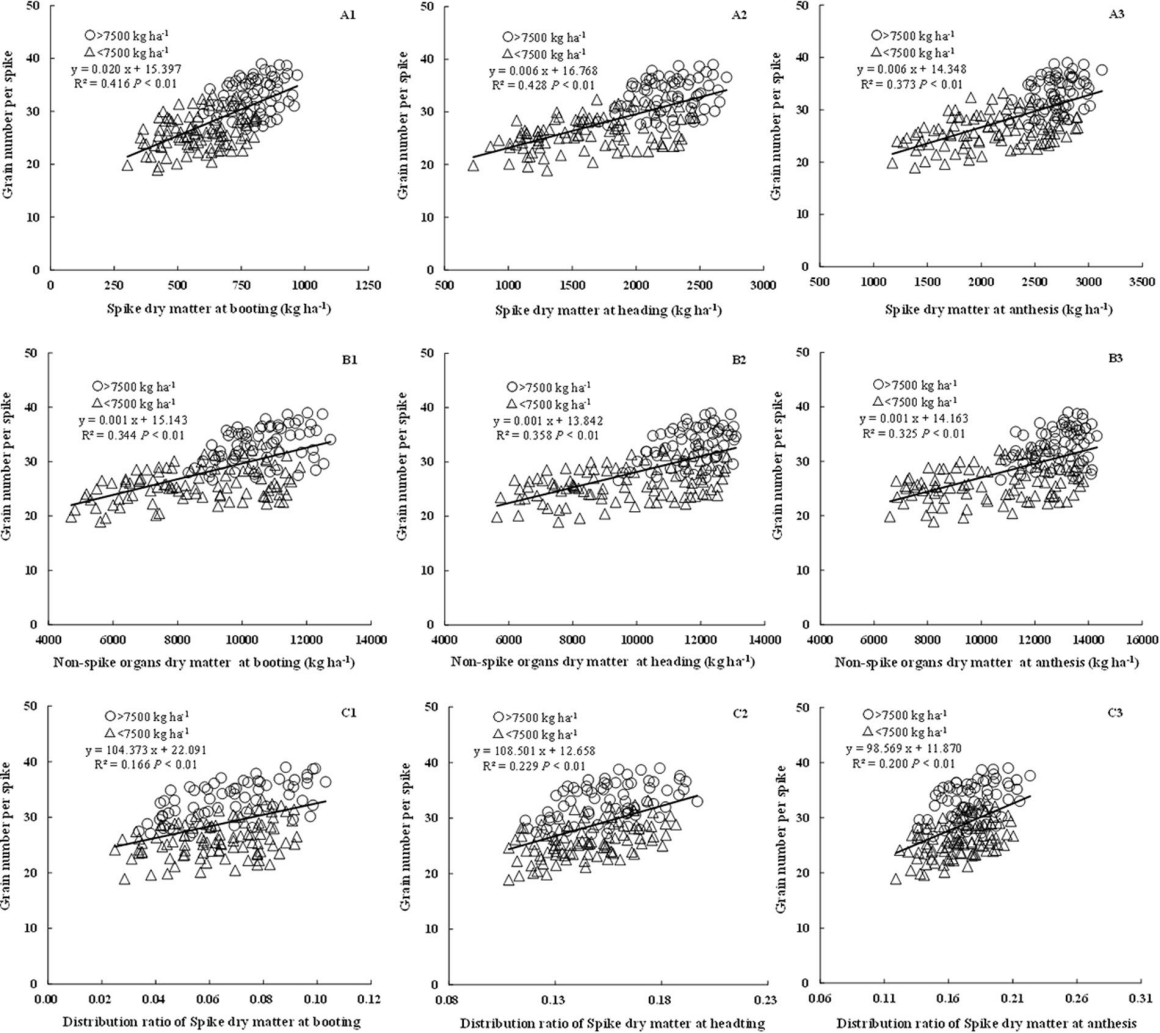
Relationship between grain number per spike and spike dry matter, non-spike organs dry matter and the distribution ratio of spikes in total aboveground dry matter during booting, heading and anthesis stages.

Comparisons of the relationships between GNs and organ nutrients at each yield level revealed a similar tendency at both higher yield levels and low yield levels. The respective datasets were therefore pooled. In this study, the aboveground organs were divided into two, spike and non-spike organs. The DM of both spike and non-spike organs showed a significantly positive relationship with GNs from booting to anthesis (R^2^ = 0.325~0.428). Furthermore, coefficients of determination for spike DM (R^2^ = 0.373~0.428) were higher than those for non-spike organs (R^2^ = 0.325~0.358) (Fig. 2A1–B3), suggesting that DM is really important factor affecting young ear development, especially spike DM. Moreover, the distribution ratio of spike DM showed a significantly positive correlation with GNs (*R*^2^ = 0.166~0.229; Fig. 2C1–C3). With growth, the demand for nutrients increases, with prior distribution to spike organs meeting the requirements of floret differentiation and grain development, and achieving higher GNs.

### Relationships between GNs and N contents of spike and non-spike organs before anthesis

**Figure 3:**
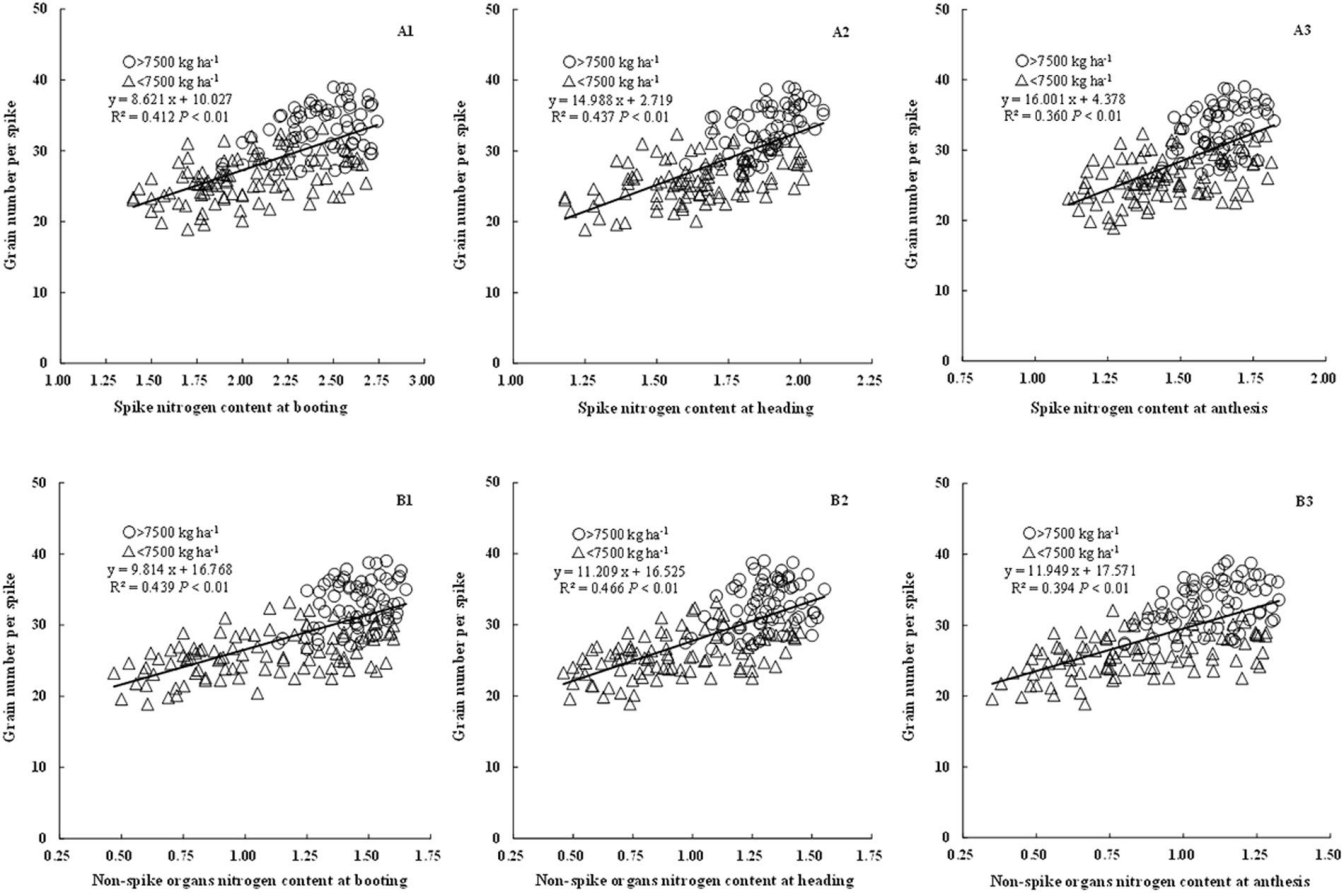
Relationship between grain number per spike and the N content of spike and non-spike organs at different phases before anthesis.

**Figure 4:**
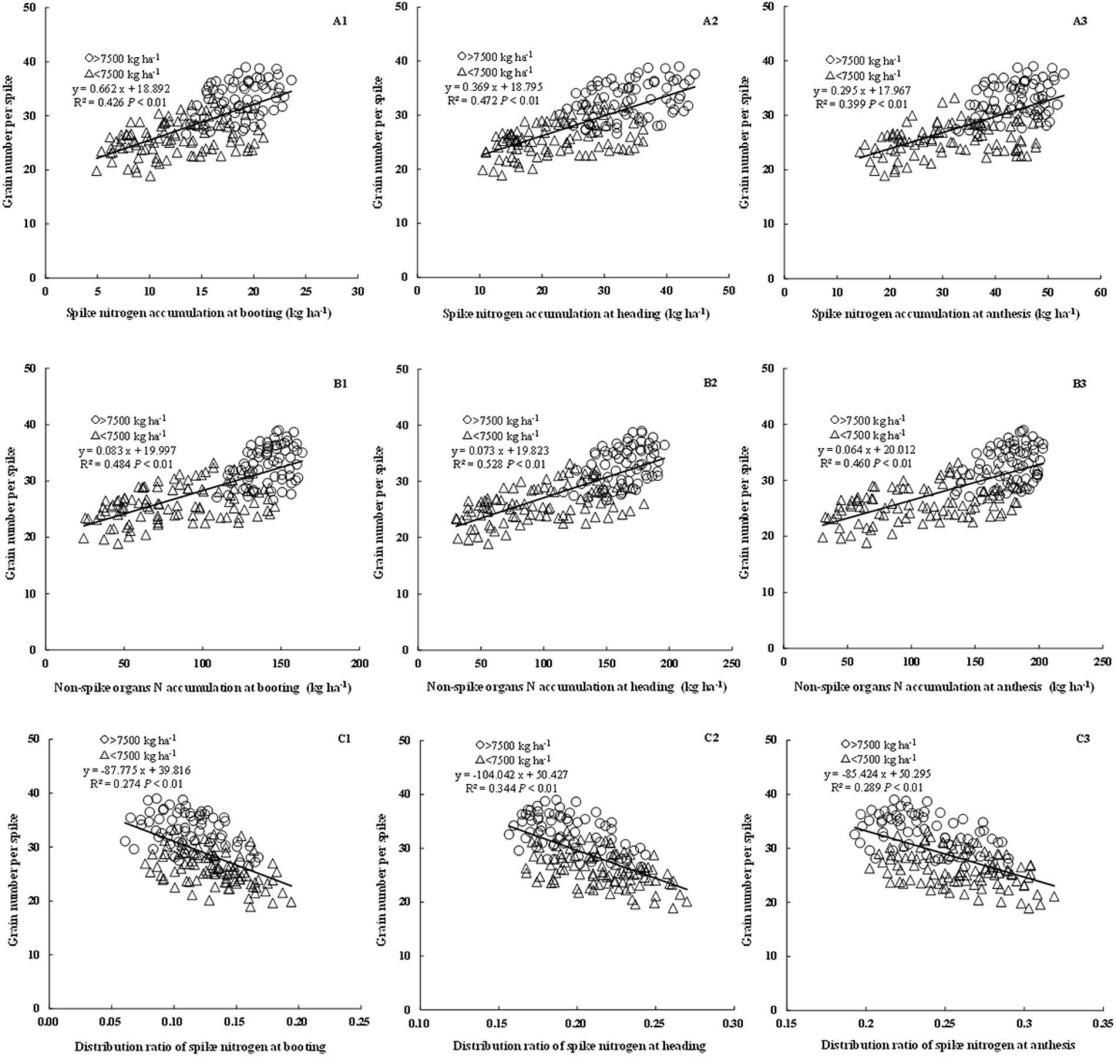
Relationship between grain number per spike and spike N accumulation, non-spike organs N accumulation and the distribution ratio of spikes in total aboveground N accumulation during booting, heading and anthesis stages.

The partitioning of aboveground N to spike and non-spike organs was also investigated from booting to anthesis. GNs was significantly positively associated with N content and accumulation in both spike and non-spike organs at all growth phases up to anthesis (R^2^ = 0.360~0.528) (Figs 3A1–B3 and 4A1–B3). Considering organ N accumulation as a product of N concentration and DM, stronger relationships were observed between GNs and N accumulation in both spike and non-spike organs (R^2^ = 0.399~0.528, Fig. 3A1–B3) compared with N concentration (R^2^ = 0.360~0.466, Fig. 4A1–B3). Compared with spike organs (R^2^ = 0.360~0.472, Figs 3A1–A3 and 4A1–A3), non-spike organs showed stronger association (R^2^ = 0.394~0.528, Figs 3B1–B3 and 4B1–B3) with GNs in terms of N content and accumulation from booting to anthesis. Moreover, the distribution ratio of spike N showed a significantly negative relationship with GNs (R^2^ = 0.274~0.344, Fig. 4C1–C3), suggesting that preferential N distribution to non-spike organs and suitable reduction in nitrogen accumulation in spike organs are conducive to increases in GNs.

There was only a negligible difference between N concentrations in terms of the correlation between GNs and spike organ nutrients (R^2^ = 0.360~0.437, Fig. 3A1–A3) and DM (R^2^ = 0.373~0.428, Fig. 2A1–A3). However, in non-spike organs, N concentration had a higher coefficients of determination (R^2^ = 0.394~0.466, Fig. 3B1–B3) with GNs compared to DM (R^2^ = 0.325~0.358, Fig. 2B1–B3). Organ N accumulation is determined by both N concentration and DM, with coefficients of determination between GNs and N accumulation (R^2^ = 0.399~0.528, Fig. 4A1–B3) higher than those with DM (R^2^ = 0.325~0.428, Fig. 2A1–B3) in both spike and non-spike organs. Moreover, a higher R^2^ seemed to be gained from non-spike than spike organs.

## Discussion

In general, increasing one or more main yield-components traits can improve yield for wheat, and the main factor usually varied in different eco-climatic regions^2,4,8,9,12^. Previous studies suggest that wheat yield improvements are primarily attributable to increases in grains number in the UK, Argentina, Mexico and Australia^31–33^, and grain weight concomitantly decreases with increasing GNa^3^. While in northern China, Zhou *et al*.^34^ found that from 1960 to 2000 improvements were primarily attributable to increased grain weight per spike. In this study, both low and higher yield levels showed significantly positive relationship between GNa and yield. Wheat yield was significantly positively related to GNa and its sub-components: GNs (R^2^ = 0.512) and SN (R^2^ = 0.629), and weakly with grain weight (R^2^ = 0.180) at low yield level. This suggests that increased yield is attributable to increases in GNa or GNs or SN, the latter being the primary factor at low yield levels. However, at higher yield levels, yield was significantly positively correlated with GNa (R^2^ = 0.240) and its sub-components GNs (R^2^ = 0.615), and poor correlation with SN (R^2^ = 0.040) and TGW (R^2^ = 0.007). These findings suggested that the function of GNs to improve yield is stronger than GNa and yield improvements are mainly attributable to marked increases in GNs at high yield levels.

Previous research suggests that yield components vary depending on plant growth conditions, with GNa being more plastic and responsive to resource availability^24,35–37^. DM production and spike weight during period of spike growth are really important components in enhancing GNa^24,35,38,39^. Arduini *et al*.^40^ reported that higher aboveground DM at heading resulted in higher remobilization of resources to the developing grains, promoting floret development and grain set. In our study, GNs showed a significant relationship with aboveground DM from jointing to anthesis. Related research confirms that crop growth rate (aboveground DM per unit area per day) during the critical period before anthesis is positively associated with spikelet and grain number^41^. In our study, the rate of aboveground DM production was strongly related to GNs during stem elongation stage from jointing to anthesis. During this period of young ear differentiation, higher DM accumulation rate led to more DM accumulation at anthesis, which was beneficial to increasied GNs. In the study region, wheat growth starts early, reaching an exuberant rate in spring (March to April), resulting in high vegetative mass and a superior canopy structure, thereby increasing radiation interception and radiation-use efficiency (RUE). It has been suggested that increased RUE before anthesis is directly related to increases in GNa^31,41,42^. Moreover, research suggests that improved total GNa is largely associated with more grains per spikelet in both wheat and rice^43,44^, and strongly positively related to spike dry weight at flowering^20,45,46^. In the present study, strong relationships between GNs and spike DM were observed from booting to anthesis. Furthermore, a significant relationship between GNs and non-spike DM was observed at three stages consistent with previous findings^19,35^. Overall, the coefficients of determination between spike DM and GNs were greater than that with non-spike DM. Our results also suggest that distribution ratio of spike DM is significantly correlated with GNs at different growth stages, with increased partitioning of assimilates to spike organs increasing the GNs. Compared to non-spike organs, spike DM (both the accumulative amount and distribution ratio) is therefore more beneficial to GNs.

N is an essential element of plant growth and one of the main limiting factors of crop production^25^. Rational application of N fertilizer can promote the synthesis of chlorophyll, increase leaf area and improve photosynthesis during crop growth^47^. When deficient, aerial DM and / or aerial N content decreases, thereby decreasing DM partitioning to the spikes and subsequent GNa in both wheat and barley^27,48^. N nutrition strongly affects N absorption and distribution in both spike and non-spike organs. Pre-anthesis spike N can be considered the result of partitioning of aboveground N accumulation between spike and non-spike organs. It was previously revealed that spike N content at anthesis is strongly related to grain number^27,28,49^. In our study, GNs was strongly related to spike N concentration and accumulation during spike growth (R^2^ = 0.360~0.472). Moreover, compared to spike organs, N concentration and accumulation in the non-spike organs was more strongly correlated with GNs before anthesis (R^2^ = 0.394~0.528). Increased partitioning of N to non-spike organs such as the leaf and stem is therefore conducive to increased green leaf area and physiological activities that improve radiation interception, RUE and DM production^31,42,50^, which would help to provide adequate nutrition for floret development and grain formation. Analysis of N distribution between spike and non-spike organs further revealed that GNs was significantly negatively related to the N distribution ratio of the spikes. This phenomenon was most likely caused by the balance between plant carbon(C) and nitrogen (N) metabolism, with a decreased proportion of carbon - nitrogen in non-spike organs contributing to the transportation of photosynthetic products to the spikes^51^. Qiu *et al*.^52^ observed that C/N value increased from booting to grain filling stage in spike organ, and the C/N value in spike organ was higher than that in non-spike organs in wheat. This indicated that more transported photosynthate to spike organ and much allocated N in non-spike organs would be conducive to promoting floret development to grain during spike growth stages. Furthermore, more N distribution in non-spike organs such as stem and leaves was beneficial to improve photosynthetic enzyme activities and photosynthesis, and subsequently produce more photosynthate^53^. Previous studies found that higher photosynthate transported to spike organ from non-spike organs was conducive to spike development, and the number of surviving florets and final grains were highly related to the proportion of photosynthate assimilates partitioned to the spikes^54,55^. These suggest that although spike N concentration and accumulation are beneficial to grain formation, an increased N distribution ratio in non-spike organs, is more beneficial to increased GNs.

GNs is a sub-component of GNa and certainly associated with GNa. Conxita *et al*.^56^ found that GNs could explain the improvement of GNa well. In our experiments, the correlation coefficient (r) between GNs and GNa reached 0.84 (*P* < 0.01). Previous studies have examined the role of plant N and DM in determining GNa or GNs. For GNa, Abbate *et al*.^28^ revealed that GNa was more closely related to spike N than spike DM accumulation. In contrast, Prystupa *et al*.^23^ revealed that GNa was more closely related to spike DM accumulation than spike N content. For GNs, Demotes-Mainard *et al*.^27^ found that GNs was related to both spike N and DM. Qiu *et al*.^52^ reported that GNs was more closely related to spike DM. In our study, we mainly focused on GNs. The relationship between GNs and spike DM (R^2^ = 0.373~0.428) was only slightly different compared with that between spike N concentration (R^2^ = 0.360~0.437). However, the N concentration and N accumulation in non-spike organs were both more strongly correlated with GNs compared to non-spike DM. The above differences may be due to differences in the wheat cultivar or organs examined, and thus, further research is needed to confirm the most important component in terms of GNs. This study provides a good theoretical basis for wheat production practice, further studies are now required to determine how to better promote the development of florets and grain setting.

## Conclusions

The major factors affecting yield improvements showed differences under different yield levels in the Huang-Huai Plain. Improvements in yield at low yield of <7500 kg ha^−1^ were attributed to increases in GNs and SN, the latter being the primary factor. Under a higher yield of >7500 kg ha^−1^, increased yield was mainly dependent on marked increases in GNs. GNs was significantly related to above-ground DM and DM accumulation from jointing to anthesis (stem rapidly elongate), higher DM growth rate results in an increase in DM accumulation at anthesis, which is highly beneficial to increased GNs and a subsequent increase in yield. For pre-anthesis nutrient (DM and N) accumulation and distribution to different organs, GNs demonstrated strong relationships with both spike and non-spike DM and N. Spike DM (both accumulative amount and distribution ratio) was more beneficial to improvements in GNs than non-spike organs. While spike N distribution ratio shows a negative correlation with GNs, and increased N accumulation and distribution ratio to non-spike organs were more conducive to improved GNs. Thus, nutrient production and distribution differ between non-spike and spike organs in terms of improvements in GNs, with increased N allocation in non-spike organs contributing to the transportation of photosynthetic products to the spikes. Generally recognizing that improved spike DM production is therefore more beneficial in promoting floret development into kernels. So, accelerated DM accumulation in the spring and improved partitioning of N to non-spike organs and DM to spike organs via cultivation management during jointing to anthesis would benefit GNs, thereby increasing grain yield.

## Materials and Methods

### Experimental site and production situation

Two field experiments were carried out with two cultivars Yumai 49–198 and Aikang58 at three sites from October 2012 to June 2015 in the Huang-huai plain, China: Shangshui (114°29′E, 33°32′N), Kaifeng (114°37′E, 34°44′N) and Wenxian (112°99′E, 34°92′N), located in southern, central and northern Henan Province, respectively. The cultivar Yumai 49–198 and Aikang 58 both are multi spike cultivars and widely planted in this experimental area. Both the two cultivars have characters of strong tillering ability (6–8 tillers per plant at jointing stage; 3–5 spikes per plant at maturity), high lodging resistance, cold resistance and high yield potentiality in field production. The yield levels of this region increased from 3000 kg ha^−1^ (1950s) to 5500 kg ha^−1^ (1980s) and now above 7500 kg ha^−1^ (after 2010 year)^57,58^. In addition, for analyzing the 161 wheat cultivars passed the examination and approval in Henan Province from 2001 to 2015, the yields of 58.3% cultivars exceed 7500 kg ha^−1^. 7500 kg ha^−1^ is classified as a higher yield standard to analyze the approaches to higher yield under different yield levels in this region^58^. The experimental sites have a warm temperate semi-humid continental monsoon climate, which result in a long tillering stage (110~120 d) and young ear differentiation period (160~170 d). Specific properties of plough layer soil and meteorological conditions during wheat growing period are shown in Table 3. The preceding crop grown in all three experimental sites was maize, with the straw returned to the field by machine after harvest.

**Table 3:** Basic physicochemical properties of the study plots at a soil depth of 0–40 cm and meteorological data during the winter wheat growing period.

Items		2012–2013			2013–2014			2014–2015		
		Shangshui	Kaifeng	Wenxian	Shangshui	Kaifeng	Wenxian	Shangshui	Kaifeng	Wenxian
CPE	PH	6.05	7.69	7.10	7.81	8.13	8.27	8.07	8.39	8.40
	TN	0.90	0.75	0.70	0.95	0.80	0.89	1.25	0.96	0.93
	AEN	60.76	49.38	89.00	90.35	74.70	93.90	110.00	85.43	100.54
	OM	15.70	12.78	12.05	16.68	12.96	12.57	18.47	14.86	13.93
	APP	5.56	10.70	13.93	12.94	12.72	12.56	27.28	13.13	16.36
	APS	138.92	166.52	205.55	116.88	136.26	190.59	124.67	164.25	191.50
WNE	PH	5.95	7.10	7.40	7.50	8.14	8.35	7.84	8.41	8.30
	TN	0.88	0.65	0.71	0.89	0.71	0.75	0.92	0.74	0.79
	AEN	65.59	45.39	69.00	75.12	52.69	78.34	83.28	84.03	92.4
	OM	14.80	13.10	12.60	16.45	12.52	12.40	17.78	14.99	14.07
	APP	5.87	11.50	12.90	12.89	12.62	12.06	23.74	7.93	19.49
	APS	144.82	135.50	168.50	119.83	131.74	158.28	138.33	162.94	187.92
Texture		LCBS	Clay	FACS	LCBS	Clay	FACS	LCBS	Clay	FACS
TPP		205.10	189.60	170.00	219.00	120.30	227.00	196.60	186.30	178.00
TEAT		2438.06	2260.71	2408.28	2668.17	2442.57	2658.61	2648.16	2691.10	2632.30
TPAR		2510.97	2292.12	2031.58	2581.30	2358.21	1987.73	2574.53	2364.14	2085.71
GD		230	237	233	227	236	234	223	232	222

Notes: CPE: Cultivation patterns experiments; WNE: Water-nitrogen mode experiments; TN: Total N (g kg^−1^); AEN: Alkaline extractable N; OM: Organic matter (g kg^−1^); APP: Available phosphorus (mg kg^−1^); APS: Available potassium (mg kg^−1^); LCBS: lime concretion black soil; FACS: fluvo-aquic clay soil; TPP: Total precipitation (mm); TEAT: Total effective accumulated temperature when averaged daily temperature >0 °C during growth period (°C); TPAR: Total photosynthetic active radiation (MJ m^−2^); GD: Growth days.

## Experimental Design

### Cultivation patterns experiment

Four cultivation patterns were tested using the wheat cultivar Yumai 49–198 as follows: local traditional cultivation pattern (TCP); optimized cultivation pattern in comparison with TCP (OCP); super high yielding cultivation pattern (SHP); and high yielding high efficiency pattern (HEP). In all cases, the date of sowing was Oct. 12~17. Under TCP, rotary tillage (about 15 cm) was carried out before sowing and no rolling was carried out after seeding. Irrigation was applied after sowing and during returning green stage. Equal row spacing of 20 cm and a large sowing quantity (225 kg ha^−1^) of seeds were applied. Under OCP, SHP and HEP, mechanical deep ploughing (over 25 cm deep) was adopted, with multiple rotary harrowing and rolling after seeding. Irrigation was applied after sowing and during jointing stage. Equal row spacing of 20 cm was adopted in OCP, and of 17 cm in SHP and HEP. A sowing quantity of 150 kg ha^−1^ was applied in OCP, and 120 kg ha^−1^ in both SHP and HEP. Irrigation was carried out, and organic, microelement and phosphate and potassium fertilizers applied before sowing (Table 4). Under TCP, all of N, phosphate (P) and potassium (K) fertilizers were applied before sowing as a base fertilizer. Under OCP and SHP, 50% of N fertilizer and all of P and K fertilizers were applied before sowing, with the remaining 50% N fertilizer applied at jointing stage. Under HEP, 40% of N fertilizer and 70% of P and K fertilizers were applied before sowing, with the remaining 60% N fertilizer and 30% P and K fertilizers applied at jointing stage. In addition, all of the organic and microelement fertilizers were applied before sowing. All four treatments followed a randomized block design with three replications, each plot 165 m^2^ (15 m in length and 11 m wide).

**Table 4:** Irrigation and fertilization management of the different cultivation patterns during the field experiments.

Treatment	Rate of fertilizer application (kg ha^−1^)					Irrigation amount and stage (m^3^ ha^−1^)			
	N	P_2_O_5_	K_2_O	ZnSO_4_	Organic fertilizer	Soil moisture water	Turning Green water	Jointing water	Blossom filling water
TCP	225	75	60	0	0	900	600	0	900
OCP	180	75	60	0	0	600	0	900	750
SHP	300	150	150	15	1500	600	0	900	900
HEP	240	90	90	15	1500	600	0	900	750

Notes: TCP: Local farmers’ traditional cultivation pattern; OCP: Optimized cultivation pattern in comparison with TCP; SHP: Super high yield cultivation pattern; HEP: High yield high efficiency pattern.

### Water-nitrogen mode experiments

Water-nitrogen mode experiments were carried out using the wheat cultivar Aikang 58 (sowing quantity was 150 kg ha^−1^), sown on Oct 12~17 at Shangshui and Wenxian. The experimental design in Shangshui consisted of a combination of two irrigation regimes (no irrigation and single irrigation at jointing stage (750 m^3^ ha^−1^), respectively) and four N rates (0,180, 240 and 300 N kg ha^−1^, respectively). In Wenxian, the design consisted of three irrigation regimes (no irrigation; single irrigation at the jointing stage (750 m^3^ ha^−1^), and irrigation at jointing plus anthesis stages (750 m^3^ ha^−1^ each), respectively) and four N rates (as above). As base fertilizers, 50% N fertilizer (Wenxian) or 70% N fertilizer (Shangshui) was applied with, all of P (100 kg P ha^−1^) and K (150 kg K ha^−1^) fertilizers before sowing. The remaining N fertilizer was applied at jointing stage. The experimental plots were arranged in a split-plot design with three replicates, each plot 40.6 m^2^ (7 m in length and 5.8 m in width). The main plot was assigned to the irrigation regime and subplots to the N rate.

## Sampling and Measurements

### Dry matter

Aboveground DM was measured at different growth stages (wintering, turning green, jointing, booting, heading and anthesis). Twenty plants were randomly sub-sampled and divided into stems and leaves during wintering to jointing stages, and into stems, leaves (non-spike organs: stems + leaves) and spike organs during booting to anthesis stages. All samples were oven dried at 65 °C and DM weighed. The distribution ratio of spike DM was calculated as the ratio of spike DM accumulation to total above-ground DM.

### Nitrogen content

N content was determined using the Kjeldahl method for each of the organs for which DM was determined during spike growth period (booting, heading and anthesis). Spike N accumulation was calculated as the product of spike N content and spike DM. Non-spike N accumulation was calculated as the product of non-spike N content and non-spike DM. The distribution ratio of spike N was calculated as the ratio of spike N accumulation to total N in the above-ground DM.

### Grain yield and yield components

Prior to harvest, the number of spikes per unit area was determined using fixed sampling points for 1 m’s double row for each treatment. At maturity, 80 wheat plants were sampled for determination of GNs, two samples of 500 grains were counted and weighed to calculate TGW. An area of 5 m^2^ was harvested, and the harvested samples threshed and dried for calculation of grain yield (kg ha^−1^).

Based on the above data, yield component data were obtained from experiments of different cultivation patterns and water-nitrogen modes from 2012–2015. DM and growth rate data were obtained from the experiments of cultivation patterns from 2012–2015 and water-nitrogen mode experiments from 2013–2015. Spike and non-spike data were obtained came from experiments of cultivation patterns and water-nitrogen modes from 2013–2015.

### Statistical analysis

Experimental data were analyzed and processed using Microsoft Office 2013. Correlations among physiological factors were determined using Pearson correlation analysis with SPSS 19.0, with significance at *P* < 0.05 and *P* < 0.01.

## Electronic supplementary material

Supplementary Information
