# Supplementary material for: Grain number responses to pre-anthesis dry matter and nitrogen in improving wheat yield in the Huang-Huai Plain
Source: Sci Rep. 2018 May 8;8:7126. doi: 10.1038/s41598-018-25608-0 (PMC5940900; doi:10.1038/s41598-018-25608-0)
Supplement: Supplementary file 1 — Supplementary Information [file 41598_2018_25608_MOESM1_ESM.docx]

Supplementary materials

**Grain number** **responses to pre-anthesis dry matter and nitrogen in improving wheat yield in the Huang-Huai** **Plain**

Jianzhao Duan ^a^, Yapeng Wu ^a^, Yi Zhou ^a^, Xingxu Ren ^a^, Yunhui Shao ^b^, Wei Feng ^a*^, Yunji Zhu ^a*^, Yonghua Wang ^a^, Tiancai Guo ^a^

^a^ National Engineering Research Centre for Wheat, State Key Laboratory of Wheat and Maize Crop Science, Henan Agricultural University, #62 Nongye Road, Zhengzhou, Henan 450002, PR China

^b^ Wheat Research Center of Henan Academy of Agricultural Sciences, Zhengzhou, 450002, China

^*^Corresponding authors. Tel.: +86 371 63558205; fax: +86 371 63558202.

E-mail addresses: fengwei78@126.com (W. Feng), hnndzyj@126.com (Y. Zhu)

Present/permanent address: National Engineering Research Centre for Wheat, #62 Nongye Road, Zhengzhou, Henan 450002, PR China

**Supplemental Figure Legends**

Figure S1. The variation of yield and its component factors between different treatments (S1 A-E)

Figure S2. The variation of dry matter and its accumulation rates during different growth stages between different treatments (S2 A-B)

Figure S3. The variation of dry matter and nitrogen accumulation indices in spike and non-spike organs during different growth stages between different treatments (S3 A-H)

Figure S1. The variation of yield and its component factors between different treatments (S1 A-E)

S1 A

S1 B

S1 C

S1 D

S1 E

Figure S2. The variation of dry matter and its accumulation rates during different growth stages between different treatments (S2 A-B)

S2 A

S2 B

Figure S3. The variation of dry matter and nitrogen accumulation indices in spike and non-spike organs during different growth stages between different treatments (S3 A-H)

S3 A

S3 B

S3 C

S3 D

S3 E

S3 F

S3 G

S3 H
